# Mobile health app for monitoring allergic rhinitis and asthma in real life in Lithuanian MASK‐air users

**DOI:** 10.1002/clt2.12192

**Published:** 2022-09-25

**Authors:** Violeta Kvedarienė, Gabija Biliute, Gabija Didziokaitė, Loreta Kavaliukaite, Agne Savonyte, Gabija Rudzikaite‐Fergize, Roma Puronaite, Jolita Norkuniene, Regina Emuzyte, Ruta Dubakiene, Arunas Valiulis, Bernardo Sousa‐Pinto, Anna Bedbrook, Jean Bousquet

**Affiliations:** ^1^ Department of Pathology Faculty of Medicine Institute of Biomedical Sciences Vilnius University Vilnius Lithuania; ^2^ Faculty of Medicine Institute of Clinical Medicine Clinic of Chest Diseases, Allergology and Immunology Vilnius University Vilnius Lithuania; ^3^ Faculty of Medicine Vilnius University Vilnius Lithuania; ^4^ Vilnius Gediminas Technical University Vilnius Lithuania; ^5^ Faculty of Medicine Institute of Clinical Medicine Clinic of Children's Diseases Vilnius University Vilnius Lithuania; ^6^ Vilnius University Medical Faculty Institute of Clinical Medicine Clinics of Chest Diseases, Allergology and Immunology Vilnius Lithuania; ^7^ Institute of Clinical Medicine and Institute of Health Sciences Medical Faculty of Vilnius University Vilnius Lithuania; ^8^ MEDCIDS – Department of Community Medicine Information and Health Decision Sciences Faculty of Medicine University of Porto Porto Portugal; ^9^ CINTESIS@RISE ‐ Health Research Network MEDCIDS Faculty of Medicine University of Porto Porto Portugal; ^10^ ARIA and MASK‐Air Montpellier France; ^11^ Institute of Allergology Charité – Universitätsmedizin Berlin Corporate Member of Freie Universität Berlin and Humboldt‐Universität zu Berlin Berlin Germany; ^12^ Fraunhofer Institute for Translational Medicine and Pharmacology ITMP Allergology and Immunology Berlin Germany; ^13^ University Hospital Montpellier Montpellier France

**Keywords:** asthma, Lithuania, MASK‐air, mHealth, rhinitis

## Abstract

**Background:**

MASK‐air^®^ is an app whose aim is to reduce the global burden of allergic rhinitis (AR) and asthma. A transfer of innovative practices was performed to disseminate and implement MASK‐air^®^ in European regions. The aim of the study was to examine the implementation of the MASK‐air^®^ app in Lithuanian adults in order to investigate (i) the rate of acceptance in this population, (ii) the duration of app use and (iii) the evaluation of the app after its use.

**Methods:**

In a longitudinal study, Lithuanian adults with AR and/or asthma were recruited by allergists. They were informed about how to use MASK‐air^®^ and were followed closely. They were reviewed after one to 3 months to evaluate satisfaction and were asked to continue using the app.

**Results:**

Among the 149 patients recruited (37.2 ± 10.4 years), 52.4% had rhinitis alone, 42.9% had rhinitis, asthma and/or conjunctivitis multimorbidity, and 2.7% isolated asthma. According to the MASK‐air^®^ baseline questionnaire, 88.3% of patients considered that their symptoms were troublesome. Data were available for 102 (68.4%) patients. The duration of app usage in patients ranged from 1 to 680 days (median, 25–75 percentile: 54, 23.2–151 days). Forty‐two (41.1% of patients who were reviewed) patients agreed to share their opinion on MASK‐air^®^. Most users of the app were satisfied, from 46.5% thinking their allergy was treated more successfully to 90.4% recommending this app to other allergy sufferers.

**Discussion:**

When recommended by physicians, MASK‐air® was used for a longer period of time.

## 1 INTRODUCTION

Digital transformation encompasses the changes associated with the application and integration of digital technology in all aspects of human life and society. It offers new types of innovation and creativity, rather than simply enhancing and supporting traditional methods.^1^ The digital transformation of health and care will benefit people, healthcare systems and the economy. The concept of digital health includes advanced medical technologies, disruptive innovations and digital communication tools, all aiming to provide the best practice care.^2^ One of the three priorities of Directorate‐General (DG) Santé (EU) concerning the digital transformation of health and care targets the empowerment of citizens. In this regard, digital tools can be used for user feedback and person‐centred care (https://ec.europa.eu/digital‐single‐market/en/news/transformation‐health‐and‐care‐digital‐single‐market‐gaining‐more‐support). Such tools include, among others, mobile health apps, which can be a valuable source of real‐world data.

MASK‐air^®^ (Mobile Airways Sentinel networK), an app of the Phase 3 Allergic Rhinitis and its Impact on Asthma (ARIA) initiative,^3^, ^4^ aims to reduce the global burden of allergic rhinitis (AR) and asthma multimorbidity, giving the patient and the healthcare professional simple tools to better prevent and manage respiratory allergic diseases. MASK‐air^®4^ is an Information and Communications Technology system centred around the patient.^4^, ^5^, ^6^, ^7^ It is operational in 27 countries and 20 languages and is freely available on Android and iOS. It includes a daily monitoring questionnaire in which patients are requested to quantify the impact of AR symptoms and to provide information on the medication used.^8^, ^9^, ^10^ However, one of its major problems is the low adherence of users: around half of the patients use the MASK‐air^®^ app only once.^7^ It is possible that adherence may improve when apps are proposed by physicians.

A transfer of innovative practices (TWINNING)^11^ was performed with the aims of (i) transferring and implementing MASK‐air^®^ in 14 different European countries (including Lithuania)^5^, ^6^ and (ii) administering the app by physicians. The “organisation transferring the innovative practice” (originator organisation) had the experience and know‐how developed in rhinitis and asthma information technology solutions. The “organisation adopting the innovative practice” (receiving/adopter organisation) received the innovative practice and implemented it in its territory.

The aim of the present study was to examine the applicability of MASK‐air^®^ in Lithuania through the TWINNING protocol,^11^ assessing the duration of use of the app and including a questionnaire on patient satisfaction obtained by the physician.

## 2 METHODS

### 2.1 Design of the study

In this study, we assessed Lithuanian patients who were taught by their physicians how to use MASK‐air®. We considered (i) their clinical and demographic features, (ii) their frequency of use of the MASK‐air® app and (iii) their opinion (in a follow‐up visit) of the app.

### 2.2 Users and settings

Over a period of 24 months, 18–60 year‐old AR and asthma patients who agreed to participate in an anonymised observational study were included in this analysis. There were no exclusion criteria. The study was performed by allergists and clinical immunologists of the outpatient clinic of the Pulmonology and Allergology Centre of Vilnius University Hospital Santaros Klinikos and of the Centre of Innovative Allergology in Lithuania.

The diagnosis of asthma or AR was based on Global Initiative for Athma (GINA)^12^ and ARIA criteria^2^ on newly‐diagnosed patients. Extra tests were not necessary, other than those of the routine allergy work‐up for patients consulting for rhinitis and/or asthma. The patients' anamnesis was collected by an allergist. Skin prick tests were performed with the clinic's regular screening panel for inhalant allergens (cat, dog, house dust mites (*D*. *pteronyssinus*, *D*. *farinae*), Alder, hazel, olive/ash, birch pollen, other tree pollen, grass pollen, parietaria pollen, cypress pollen, ragweed pollen, and other inhalant allergens (Inmunotek, SL,.Spain). For the patients who were using oral H_1_‐antihistamines, the immunoblot panel of serum‐specific IgE of inhalant allergens was performed (Euroline, Euroimmun).^13^ To confirm the diagnosis in patients with asthma, spirometry with bronchodilator or the methacholine challenge test were performed according to the GINA guidelines.

The included patients were trained to use the MASK‐air^®^ app in the clinic. An allergist, a trained student, or a resident in allergology showed each patient the MASK‐air app, teaching him/her how to add data. On the same day, the patient filled in and completed the first personal evaluation of AR and asthma symptoms, and the doctor answered any practical questions. MASK‐air^®^ collects information on patients' baseline characteristics, usual rhinitis and asthma symptoms as well as disease type (intermittent/persistent). In addition, MASK‐air® comprises a daily monitoring questionnaire assessing (i) how rhinitis and asthma symptoms impact users' lives each day and (ii) type(s) of treatment used.^7^, ^9^, ^14^

### 2.3 Ethics

The MASK‐air^®^ app is a CE1 device.^15^ MASK‐air^®^ is in line with the General Data Protection Regulation (GDPR) EU Directive 95/46/EC.^16^ The data are anonymised, including the data related to geolocalisation, using k‐anonymity.^17^ The overall international study was approved by the Ethics Committee of the Bohn‐Cologne, and the Lithuanian arm by the Ethics Committee of Vilnius City Clinical Hospital n° IS‐515/21(2.25). Users agreed to having their data analysed (terms of use).^11^

### 2.4 Follow‐up of the patients

The patients were reassessed 1–3 months after starting the app, according to the routine follow‐up of patients.

### 2.5 Outcomes

#### 2.5.1 Baseline characteristics of the patients

In the first outpatient visit, baseline asthma and rhinitis symptoms (including rhinorrhoea, sneezing, nasal congestion, nasal itching and ocular symptoms) were assessed using the MASK‐air^®^ app.^10^, ^14^ That same day, we assessed the ARIA severity score which was calculated using the four questions regarding impact on sleep, daily activities, work/school attendance and bothersome symptoms. Each of these four items were ascribed a score of 1 (“Yes”) or 0 (“No”). The total ARIA score ranged from 0 (no impairment) to 4 (severe impairment).

Patients also filled in the Control of Allergic Rhinitis and Asthma Test (CARAT) questionnaire. Control of Allergic Rhinitis and Asthma Test is a Patient‐Reported Outcome that assesses the level of control of both asthma and AR in the past 4 weeks using a single tool.^18^ It encompasses 10 questions, with the first four (CARAT Q1‐4) concerning the upper airways, and the last six (CARAT Q5‐10) the lower airways. Results are presented on a scale of 0–30, with higher values indicating better control. In a quasi‐experimental study in Greece, CARAT and MASK‐air^®^ were found to provide complementary information on AR symptom control, possibly mirroring differences in the time periods assessed by these two tools.^19^

#### 2.5.2 Duration of usage

The duration of MASK‐air® usage was assessed by determining the number of days of reporting, as estimated in previous studies.^20^

#### 2.5.3 Patients' and physicians' rating of the app

One to three months after starting the MASK‐air^®^ app, the patients were asked eight questions regarding their satisfaction. Five replies were available for each question (ranging from strong disagreement to strong agreement) (Table 1).

**TABLE 1:** Possible options of questions and answers

Questions	Answers
1. The app is user‐friendly	1. Strongly agree
2. The app is working properly	2. Agree
3. All of the information is presented in an understandable way	3. Neither agree nor disagree
4. I like the appearance of the app	4. Disagree
5. The app meets all of my expectations/needs	5. Strongly disagree
6. I like using the app	
7. Thanks to the app, my allergy is being treated more successfully	
8. I would recommend this app to someone with an allergy	

### 2.6 Size of the study

In this pilot study, all registered users were included to obtain the best possible estimates for the specified time window.

### 2.7 Analysis of the data and statistical methods

When responding to the MASK‐air^®^ daily monitoring questionnaire, it is not possible to skip any of the questions, and data are saved to the dataset only after the final answer. This precludes any missing data. Categorical variables were described using absolute and relative frequencies, and tested using the chi‐square test. Except for demographic data, a non‐Gaussian distribution was found for continuous variables, and therefore medians (and percentiles) and non‐parametric tests were used. Correction for multiple testing using the Bonferroni's correction was made when appropriate. The Spearman rank correlation test was used to measure the degree of association between two continuous variables.

## 3 RESULTS

### 3.1 Demographic characteristics of patients

The study included 149 patients ranging in age from 18 to 60 years (mean ± SD: 37.2 ± 10.4 years). There were 55.0% of women and 45.0% of men. Seventy‐eight (52.4%) patients had only AR, 55 (36.9%) were suffering from AR and asthma, 8 (5.4%) patients had AR and allergic conjunctivitis (AC), 4 (2.7%) isolated asthma and 4 (2.3%) were suffering from all three (AR, asthma and AC). Most patients (*N* = 108; 72.5%) had a bachelor or postgraduate degree and 104 (69.8%) had a full‐time job. In the study, subjects were divided into two groups of similar size. The first group consisted of 78 (52.3%) patients with a diagnosis of AR only. The second consisted of 71 (47.7%) multimorbid AR patients with a diagnosis of AR and/or asthma and/or AC. No significant differences were observed across patients' diagnoses in relation to their gender, educational level or work status. One hundred and two patients were seen at the follow‐up visit (68.4%).

For 57 (38.3%) patients, allergic sensitisation had been diagnosed with skin tests and 74 (49.7%) had positive skin‐prick tests and serum‐specific IgE to inhalant allergens. The most common allergens were house dust mites, grass, birch pollen, ragweed, wormwood and cat (Table 2 online). Eighty‐five (57.0%) subjects were polysensitised, 42 (28.2%) were monosensitised and 22 (14.8%) did not report any allergen sensitisation (Table 2 online).

**TABLE 2:** Associations between diagnosis and gender, education and work status

	Sole allergic rhinitis (*N* = 78)	Multimorbid allergic rhinitis (*N* = 71)	*p*‐value
Gender—*N* (%)			0.98
Male	35 (44.9)	32 (45.1)	
Female	43 (55.1)	39 (54.9)	
Education—*N* (%)			0.07
Secondary school or less	11 (14.1)	9 (12.7)	
College	7 (9.0)	13 (18.3)	
Bachelor	36 (46.1)	20 (28.2)	
Postgraduate	23 (29.5)	29 (40.8)	
Work status—*N* (%)			0.30
Student	12 (15.4)	4 (5.6)	
Part‐time job	6 (7.7)	5 (7.0)	
Full‐time job	53 (67.9)	54 (76.1)	
Unemployed	4 (5.1)	7 (9.9)	
Paid/sick leave	3 (3.8)	4 (5.6)	

### 3.2 Symptoms

**FIGURE 1:**
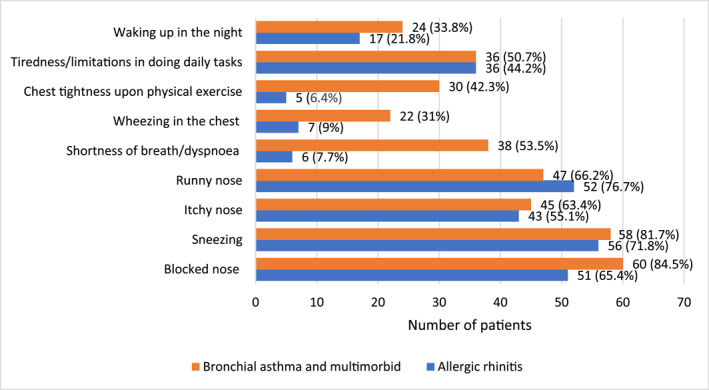
Assessment of allergic rhinitis (AR) symptoms according to the MASK‐air baseline and Control of Allergic Rhinitis and Asthma Test (CARAT) questionnaires

The most common symptoms in patients with AR alone were rhinorrhoea (76.7%), sneezing (71.8%) and blocked nose (65.4%). Patients with AR and asthma most often complained of blocked nose and sneezing (85.5%), nasal itching and rhinorrhoea (67.3%). Individuals with AR and AC complained mostly of a stuffy nose and sneezing (62.5% each) (Figure 1).

Using the Bonferroni's correction, shortness of breath (*p* < 0.001), wheezing (*p* < 0.001) and chest tightness during exercise (*p* < 0.001) were more severe in the group of patients with multimorbid rhinitis, asthma and conjunctivitis than in those with a single disease. Comparing the severity of symptoms by gender, women had more severe symptoms of blocked nose (*p* = 0.003), more frequent nasal itching (*p* = 0.002) and greater fatigue during daily work (*p* < 0.001) (Table 3 online and Figure 1). There were no significant differences in the frequency of symptoms between patients who were sensitive to one or more allergens.

**TABLE 3:** Sensitisation profile of patients

Allergen	Frequency of sensitised patients—*N* (%)
House dust mites	67 (45.0)
Cat	46 (30.9)
Dog	23 (15.4)
Alder, hazel and/or birch	59 (39.6%)
Other tree pollen	19 (12.8)
Grass	59 (39.6)
Ragweed	48 (32.2)
Other inhalant allergens	10 (6.7%)
Food allergens	8 (5.4)
None	22 (14.8)

### 3.3 Impact of allergic diseases

**FIGURE 2:**
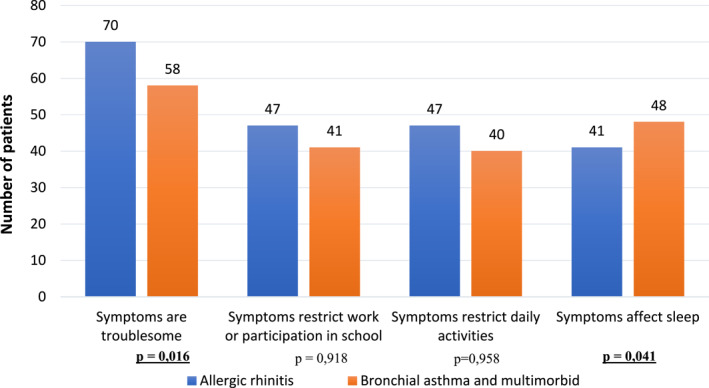
Assessment of impact of allergic rhinitis (AR) symptoms according to the MASK‐air baseline questionnaire

According to the MASK‐air^®^ baseline questionnaire, the vast majority (88.3%) of all patients considered that their symptoms were troublesome. Symptoms affected sleep in 61.8% of patients and restricted activities in 60.8% and work participation in 61.8%. Comparing the subjective assessment of symptoms by disease groups (sole AR or multimorbid AR), significant differences were observed. Multimorbid AR patients were more likely to complain of sleep disorders (*p* = 0.041). On the other hand, patients with AR alone reported their symptoms as bothersome more often than the multimorbid AR patients (*p* = 0.016). No statistical differences were found when comparing the subjective assessment of symptoms by gender (Figure 2). There were significant but weak‐to‐moderate correlations between all four outcome measures (Table 4 online).

**TABLE 4:** Comparison of the frequency of symptoms according to the existence of multimorbidity, gender and sensitisation pattern

Symptom (%)	Disease			Gender			Sensitisation		
	Sole AR	Multimorbid AR	*p*‐value	Male	Female	*p*‐value	Mono	Poly	*p‐*value
Blocked nose	67.5	83.3	0.02	63.9	84.1	0.003	66.4	62.8	0.59
Sneezing	72.7	77.5	0.48	68.7	80.2	0.09	66.2	62.9	0.62
Itchy nose	72.0	78.3	0.34	63.8	84.2	0.002	59.5	66.2	0.31
Runny nose	74.3	75.8	0.83	71.1	78.2	0.30	69.5	61.3	0.22
Shortness of breath/dyspnea	58.3	93.3	<0.001	70.2	79.0	0.12	65.1	63.4	0.76
Wheezing in the chest	66.8	84.0	<0.001	71.7	77.7	0.22	68.2	62.0	0.20
Chest tightness upon physical exercise	62.1	89.2	<0.001	72.6	77.0	0.41	63.0	64.5	0.78
Tiredness/limitations in doing daily tasks	72.0	78.3	0.34	59.7	87.5	<0.001	69.5	61.3	0.12
Waking up in the night	70.8	79.6	0.11	71.1	78.2	0.20	67.1	62.5	0.37

Abbreviations: AR, Allergic rhinitis; Mono, Monosensitisation; Poly, Polysensitisation.

### 3.4 Duration of app usage

**FIGURE 3:**
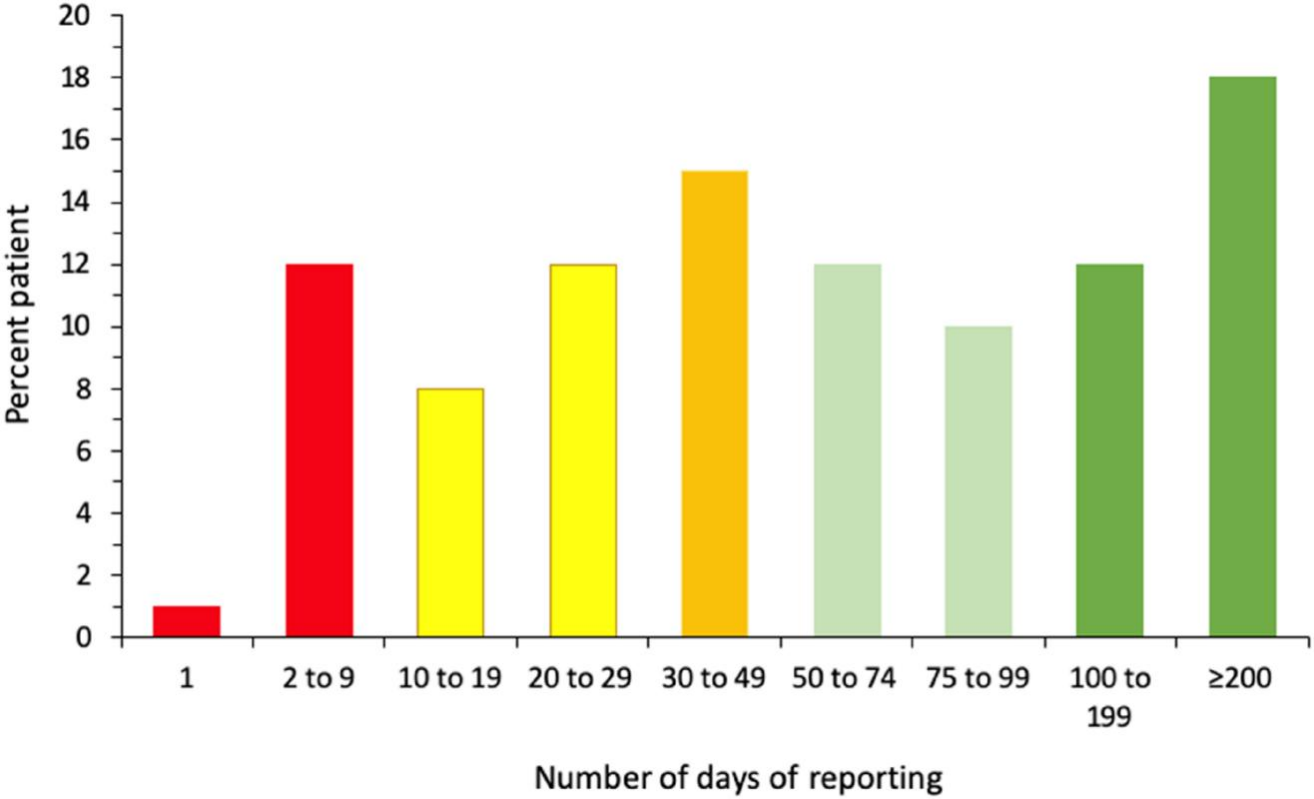
Repartition of the number of days reported by patients

Data were available for 102 (68.4%) patients. The duration of app usage in patients ranged from 1 to 680 days (median, 25–75 percentiles: 54, 23–151 days). The repartition of reported days is presented in Figure 3. Only one patient used the app once. The repartition of patients was similar in days 2–9 and days 100–199 (8%–15%). However, there was an increased frequency when the reported days were above 200 (18%).

### 3.5 Rating of the app by patients

**FIGURE 4:**
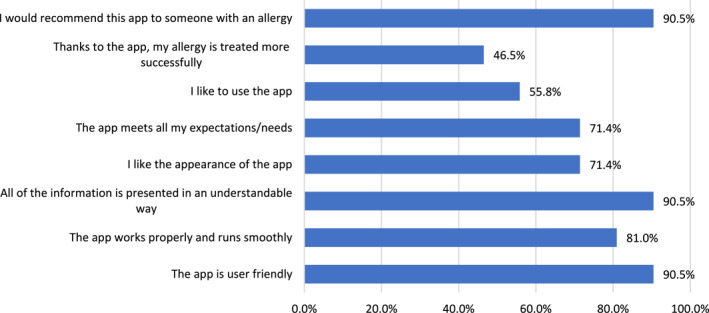
Evaluation of the MASK‐air app by patients

Forty‐two (41.1% of patients who were reviewed) of the patients who had used the MASK‐air^®^ app for over a month agreed to share their opinion on this tool. All of them had either AR alone or both AR and asthma. Women comprised 52.4% (*N* = 22) of the respondents. Most users of the app (90.47%) were satisfied with the information provided, found the app to be user‐friendly and would recommend it to another person with allergies. Less frequently, patients agreed with the statements indicating that they like to use the app and that it helps to treat allergies more successfully (55.81% and 46.51%) (Figure 4).

Younger patients were more likely to find the app user‐friendly and to recommend it to another person with allergies (*p* = 0.027), but older users were more satisfied with the design of the app (*p* = 0.042). Comparing the responses by gender, women rated the app more favourably in all aspects. They significantly more often thought that the app was user‐friendly and that the information contained was understandable (100% women vs. 80% men, *p* = 0.043). Compared by disease, patients in the sole AR group were more likely to find the app user‐friendly (*p* = 0.012).

## 4 DISCUSSION

In this study, we assessed patients with sole or multimorbid AR recruited by physicians. These patients were taught how to use the MASK‐air^®^ app. The recruited users appear to be similar to European MASK‐air^®^ users overall regarding their demographic and clinical characteristics, but they display substantially higher adherence to the app. Overall, this suggests that adherence to mobile apps may be higher when the latter are promoted by physicians and when the users are taught how to use them. Of note, this study also found that patients are overall satisfied with MASK‐air^®^ use, and the results obtained with adults are in line with a MASK‐air^®^ study on older adults in Puglia.^21^

In the present study, we found that most patients had either AR alone (52.4%) or both AR and asthma (36.9%). In the first set of analyses, we assessed AR symptoms and impact at baseline. We then compared them with a recent MASK‐air^®^ paper in which 9037 European users from 17 countries were investigated.^10^ In the former study, users had slightly fewer symptoms, particularly outside the predicted pollen season, than in the current study, and the impact was also less important. Thus, patients included in this study may have more severe presentations than overall MASK‐air^®^ users, possibly because they were recruited from an allergy clinic. However, in both studies, bothersome symptoms were found in 68% (outside the pollen season), 76% (during the pollen season) and 88% (current study) of cases (Table 5 online).

**TABLE 5:** Correlation between impact outcomes as assessed by the MASK‐air® baseline daily monitoring questionnaire

	Symptoms affect sleep	Symptoms restrict daily activities	Symptoms restrict work or participation in school
Symptoms affect sleep			
Symptoms restrict daily activities	0.305		
Symptoms restrict work or participation in school	0.352	0.576	
Symptoms are troublesome	0.348	0.340	0.363

*Note*: Values presented as Spearman correlation coefficient; *p*‐value < 0.001.

Achieving sufficient mHealth App engagement and user retention rates is a difficult task.^22^ In the latest MASK‐air^®^ analysis (December 4, 2020), there were 17,780 users for 317,000 days (mean: 17.83 days/user). In the present study, there were 10,928 days for 102 patients (mean: 107.14 days/user). Although it is difficult to compare two sets of data, users reporting MASK‐air^®^ data use the same app similarly, and the very large difference suggests that when an app is proposed by physicians (who train their patients to use it), the patients report their use for a longer period of time. This does not apply to all users, but only one patient included in this study reported a single day of MASK‐air^®^ use. The low retention rate of mobile apps is not restricted to MASK‐air^®^. Numerous factors have been identified to potentially influence engagement, and it is important to consider these in order to best overcome them.^23^, ^24^ Engagement strategies should consider usability of technology, motivating factors and the need for personal contact. In the present study, personal professional contact with physicians and a short training session on the use of the app are likely to lead to a higher retention rate (Table 6).

**TABLE 6:** Comparison of symptoms and their effects between the two studies

	Results of another study during pollen season	Results of another study outside pollen season	The results of our study
Symptoms day 1			
Itchy nose (%)	73	66	60
Sneezing (%)	61	55	77
Congestion (%)	69	65	75
Impact of symptoms day 1			
Sleep (%)	38	35	62
Daily activities (%)	45	39	61
Work/school (%)	30	26	60
Bothersome (%)	76	68	88

## 5 CONCLUSIONS

This study suggests that when mHealth apps are proposed by physicians, users report data for a longer period of time.

## AUTHOR CONTRIBUTIONS

**Violeta Kvedarienė**: Conceptualisation; Writing – review & editing. **Gabija Biliute**: Writing – Review & editing. **Gabija Didziokaitė**, **Loreta Kavaliukaite**, **Agne Savonyte**, **Gabija Rudzikaite‐Fergize**: Data collection; Writing. **Roma Puronaite**, **Jolita Norkuniene**: Statistical analysis; Supervision; Methodology. **Regina Emuzyte**, **Ruta Dubakiene**, **Arunas Valiulis**: Writing – review & editing. **Bernardo Sousa‐Pinto**: review & editing. **Anna Bedbrook**: Writing – review & editing; Visualisation. **Jean Bousquet**: Conceptualisation; Methodology; Writing – review & editing; Visualisation.

## CONFLICTS OF INTEREST

Jean Bousquet reports personal fees from Chiesi, Cipla, Hikma, Menarini, Mundipharma, Mylan, Novartis, Purina, Sanofi‐Aventis, Takeda, Teva, Uriach, other from KYomed‐Innov, VK reports non‐financial support from BerlinCHemie Menarini, Norameda. The other authors have no COIs to declare.

## References

[1] Bedard A , Anto JM , Fonseca JA , et al. Correlation between work impairment, scores of rhinitis severity and asthma using the MASK‐air((R)) App. Allergy. 2020;75(7):1672‐1688. 31995656 10.1111/all.14204

[2] Mesko B , Drobni Z , Benyei E , Gergely B , Gyorffy Z . Digital health is a cultural transformation of traditional healthcare. mHealth. 2017;3:38. 10.21037/mhealth.2017.08.07 29184890 PMC5682364

[3] Bousquet J , Khaltaev N , Cruz AA , et al. Allergic rhinitis and its impact on asthma (ARIA) 2008 update (in collaboration with the world health organization, GA(2)LEN and AllerGen). Allergy. 2008;63((Suppl 86)):8‐160. 18331513 10.1111/j.1398-9995.2007.01620.x

[4] Bousquet J , Hellings PW , Agache I , et al. ARIA 2016: care pathways implementing emerging technologies for predictive medicine in rhinitis and asthma across the life cycle. Clin Transl Allergy. 2016;6:47. 28050247 10.1186/s13601-016-0137-4 PMC5203711

[5] Bourret R , Bousquet JJM , Mercier TC , et al. MASK rhinitis, a single tool for integrated care pathways in allergic rhinitis. World Hosp Health Serv. 2015;51(3):36‐39. 26571642

[6] Bousquet J , Schunemann HJ , Fonseca J , et al. MACVIA‐ARIA sentinel networK for allergic rhinitis (MASK‐rhinitis): the new generation guideline implementation. Allergy. 2015;70(11):1372‐1392. 26148220 10.1111/all.12686

[7] Bousquet J , Arnavielhe S , Bedbrook A , et al. MASK 2017: ARIA digitally‐enabled, integrated, person‐centred care for rhinitis and asthma multimorbidity using real‐world‐evidence. Clin Transl Allergy. 2018;8:45. 30386555 10.1186/s13601-018-0227-6 PMC6201545

[8] Bousquet J , Anto JM , Bachert C , et al. ARIA digital anamorphosis: digital transformation of health and care in airway diseases from research to practice. Allergy. 2021;76(1):168‐190. 32512619 10.1111/all.14422

[9] Bousquet J , Bedbrook A , Czarlewski W , et al. Guidance to 2018 good practice: ARIA digitally‐enabled, integrated, person‐centred care for rhinitis and asthma. Clin Transl Allergy. 2019;9:16. 30911372 10.1186/s13601-019-0252-0 PMC6413444

[10] Bedard A , Basagana X , Anto JM , et al. Treatment of allergic rhinitis during and outside the pollen season using mobile technology. A MASK study. Clin Transl Allergy. 2020;10(1):62. 33298191 10.1186/s13601-020-00342-x PMC7726888

[11] Bousquet J , Agache I , Aliberti MR , et al. Transfer of innovation on allergic rhinitis and asthma multimorbidity in the elderly (MACVIA‐ARIA) ‐ EIP on AHA twinning reference site (GARD research demonstration project). Allergy. 2018;73(1):77‐92. 28600902 10.1111/all.13218

[12] Reddel HK , FitzGerald JM , Bateman ED , et al. GINA 2019: a fundamental change in asthma management: treatment of asthma with short‐acting bronchodilators alone is no longer recommended for adults and adolescents. Eur Respir J. 2019;53(6):1901046. 10.1183/13993003.01046-2019 31249014

[13] Huang Z , Zou X , Chen H , et al. Identifying potential Co‐sensitization and cross‐reactivity patterns based on component‐resolved diagnosis. Int Arch Allergy Immunol. 2020;181(2):81‐93. 10.1159/000504320 31770759

[14] Bousquet J , Caimmi DP , Bedbrook A , et al. Pilot study of mobile phone technology in allergic rhinitis in European countries: the MASK‐rhinitis study. Allergy. 2017;72(6):857‐865. 10.1111/all.13125 28072463

[15] Council directive 93/42/EEC of 14 June 1993 concerning medical devices. 1993L0042 — EN — 11.10.2007 — 005.001 — 1. https://eur‐lexeuropaeu/LexUriServ/LexUriServdo?uri=CONSLEG:1993L0042:20071011:EN:PDF. 1993.

[16] Directive 95/46/EC of the European Parliament and of the Council of 24 October 1995 on the protection of individuals with regard to the processing of personal data and on the free movement of such data ; Off J Eur Communities, L281, 31; 23. 1995.

[17] Samreth D , Arnavielhe S , Ingenrieth F , et al. Geolocation with respect to personal privacy for the Allergy Diary app ‐ a MASK study. World Allergy Organ J. 2018;11(1):15. 10.1186/s40413-018-0194-3 30061979 PMC6048852

[18] Fonseca JA , Nogueira‐Silva L , Morais‐Almeida M , et al. Validation of a questionnaire (CARAT10) to assess rhinitis and asthma in patients with asthma. Allergy. 2010;65(8):1042‐1048. 10.1111/j.1398-9995.2009.02310.x 20121755

[19] Mitsias DI , Dimou MV , Lakoumentas J , et al. Effect of nasal irrigation on allergic rhinitis control in children; complementarity between CARAT and MASK outcomes. Clin Transl Allergy. 2020;10(1):9. 10.1186/s13601-020-00313-2 32190296 PMC7068957

[20] Bedard A , Basagana X , Anto JM , et al. Mobile technology offers novel insights on control and treatment of allergic rhinitis. The MASK study. J Allergy Clin Immunol. 2019. 10.1016/j.jaci.2019.01.053 30951790

[21] Ventura MT , Giuliano AFM , Buquicchio R , et al. Implementation of the MASK‐air® app for rhinitis and asthma in older adults: MASK@Puglia pilot study. Int Arch Allergy Immunol. 2022;183(1):45‐50. 10.1159/000518032 34569536

[22] Bousquet J , Ansotegui IJ , Anto JM , et al. Mobile technology in allergic rhinitis: evolution in management or revolution in health and care? J Allergy Clin Immunol Pract. 2019;7(8):2511‐2523. 10.1016/j.jaip.2019.07.044 31445223

[23] Druce KL , Dixon WG , McBeth J . Maximizing engagement in mobile health studies: lessons learned and future directions. Rheum Dis Clin N Am. 2019;45(2):159‐172. 10.1016/j.rdc.2019.01.004 PMC6483978 30952390

[24] Simblett S , Matcham F , Siddi S , et al. Barriers to and facilitators of engagement with mHealth technology for remote measurement and management of depression: qualitative analysis. JMIR mHealth Uhealth. 2019;7(1):e11325. 10.2196/11325 30698535 PMC6372936

